# Health impact assessment of PM2.5 from uncovered coal trains in the San Francisco Bay Area: Implications for global exposures

**DOI:** 10.1016/j.envres.2024.118787

**Published:** 2024-03-29

**Authors:** Bart Ostro, Yuanyuan Fang, Marc Carreras Sospedra, Heather Kuiper, Keita Ebisu, Nicholas Spada

**Affiliations:** a University of California, Air Quality Research Center, Davis, USA; b Independent Consultant, USA; c University of California, Irvine, USA; d California Office of Environmental Health Hazard Assessment, USA

**Keywords:** PM2.5, Coal, Dust, Trains, Mortality, Asthma

## Abstract

Coal generates almost 40% of the world’s electricity with 80 countries throughout the world using coal power. An inherent part of this generation is the rail transport of coal in uncovered cars, often up to a mile long. Existing studies document the subsequent increments of PM2.5 to the near-rail populations, which typically include a large number of economically disadvantaged residents and/or people of color. However, to date there is no assessment of the health implications of this stage in the use of coal. The present study quantifies such impacts on a region in the San Francisco Bay Area. The analysis shows important effects on mortality, hospitalization for cardiovascular and respiratory disease, asthma exacerbation, work loss, and days of restricted activity. Several of these outcomes exhibited a one to six percent increase over baseline. As such, it delineates the implications for the global effects of the transport of coal.

## 1. Introduction

Coal generates almost 40% of the world’s electricity and there are now 80 countries using coal power (Energy Institute, 2023; International Energy Agency, 2023). A recent study following the Medicare population over 20 years estimated that during that period, electric generation from coal was responsible for 460,000 deaths in the United States (Henneman et al., 2023). Another study estimated approximately five million excess deaths per year globally attributable to ambient air pollution from fossil fuel use (Lelieveld et al., 2023) making fossil fuels the largest source of ambient PM_2.5_-related mortality. Among the fossil fuels, coal is the largest source of this mortality (Vohra et al., 2021; McDuffie et al., 2021). However, the health impact associated with an integral aspect of this power generation - the rail transport of coal to power and export facilities throughout the United States and globally - remains unexplored in the existing literature.

Trains transport coal throughout the United States and the rest of the world in uncovered cars, often extending up to a mile long. The transportation of coal has been shown to significantly increase ambient fine particulate matter (PM2.5) (Ostro et al. 2023a, 2023b; Jaffe et al., 2015; Akaoka et al., 2017). Further, studies have demonstrated that ambient PM2.5 concentrations of passing coal cars are greater than those generated by freight or passenger trains, and that these emissions can reach residential communities (Ostro et al., 2023a; Jaffe et al., 2015). The population-level effect of coal transport by rail is significant, in part because this activity is widespread; nearly 70% of all coal is transported by rail in the US, and this transport constitutes 14% of all rail freight activity (Freightwaves, 2019; U.S. Energy Information Administration, 2023). Further, this conveyance, which occurs globally, often involves a traverse through densely populated urban settings inhabited by communities of color and/or those who are economically disadvantaged. This indicates that any associated emissions constitute a significant public health and environmental justice concern.

Exposure to PM2.5 is causally associated with a wide range of adverse health effects. These effects range from minor respiratory symptoms or work loss to severe consequences including asthma and heart attacks, adverse birth outcomes, emergency room visits and hospitalizations, neurological and cognitive impacts, and premature mortality. Studies have indicated that these effects occur from both chronic (lasting a year or more) and acute (daily or even hourly) exposures (U.S, EPA, 2023a,b). Further, studies have failed to identify a threshold concentration, as effects have been found at concentrations far below the current U.S. air quality standard of 9 μg/m^3^ (World Health Organization, 2021; Crouse et al., 2020). As a result, exposure to PM2.5 is the fourth leading global risk factor for mortality (Fuller et al., 2022). Of additional relevance, combusted coal, as a fossil fuel, is a major contributor to greenhouse gases and when these embedded emissions are taken into account, the railroads produced 16.5 percent of total U.S. carbon pollution (Meyer, 2019).

Given the prevalence of the global transit of coal in open-topped coal cars and the subsequent population exposure, it is important to quantify likely associated health impacts using the formal approach of a health impact assessment (HIA). Using artificial intelligence to identify passing coal trains (versus freight or passenger trains), Ostro et al. (2023a,b) estimated the subsequent increments in PM2.5, forming the foundations of this present health impact analysis.

Below, we quantify the health impacts from PM2.5 emitted from the passage of coal trains for communities living near the rail lines. Estimates are also provided for race-specific relative differences in annual PM2.5 exposures from the passing trains. Because this population is likely to be predominately more vulnerable to adverse air pollution impacts based on their income, education, race and ethnicity, we describe the environmental justice implications as well (Colmer et al., 2020; Tessum et al., 2021).

## 2. Study population

**Fig. 1.**
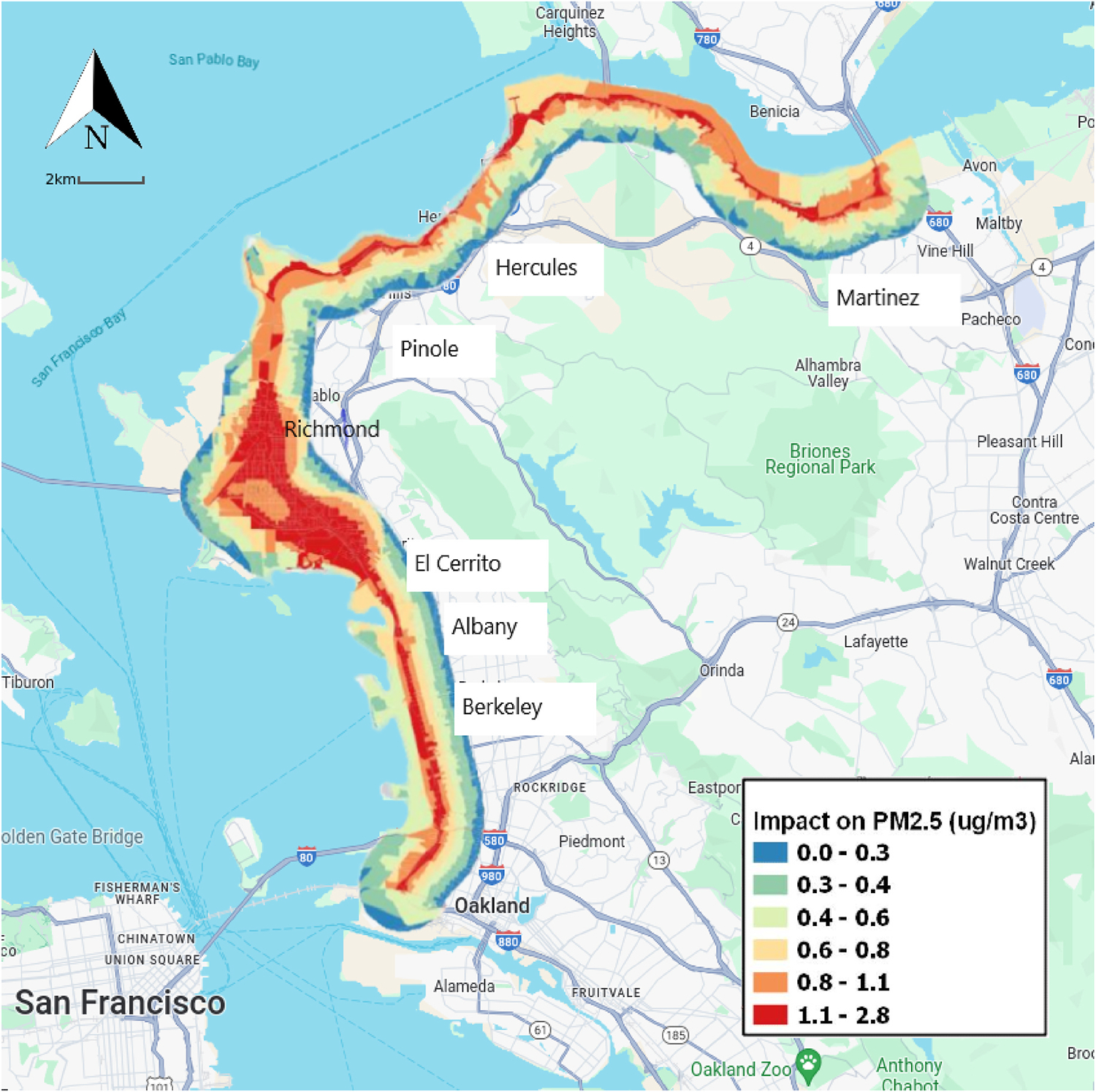
Study Area with Estimated PM2.5 Concentrations associated with 2.1 μg/m^3^ Increase in the Peak of the Annual Average Increment..

The study area includes cities in the East Bay region of the San Francisco Bay Area, California, approximately 12 miles east of San Francisco itself (Fig. 1). The study area comprises parts of several large cities including Oakland, Richmond, and Berkeley and our analysis stretches from Oakland to Martinez. As such, the present assessment focuses on populations proximate to the rail corridor and export facilities pertaining to an existing coal export terminal in Richmond, California and a proposed coal export terminal farther down the rail line in the West Oakland neighborhood of the City of Oakland (Fig. 1). Fig. 1 also shows the path of the train line and the associated PM2.5 exposures.

The exposed area has a racially and ethnically diverse population, including Hispanic (35%), White (26%), Black (22%), Asian (17%) and Native American and Pacific Islands (<1%) residents. Both the cities of Richmond and Oakland have areas with a high degree of low-income residents and significant co-morbidities. These areas tend to have higher rates of mortality, asthma emergency room visits, cardiovascular disease, unemployment, and lower educational attainment than other areas of their respective counties (BAAQMD, 2018).

## 3. Methodology

The HIA quantifies changes in the incidence of adverse health outcomes resulting from changes in population exposure to PM2.5. HIAs have been used in hundreds of studies around the globe and are a well-established approach for estimating the past or future impacts associated with changes in air pollution. The software Environmental Benefits Mapping and Analysis Program (BenMAP) has been widely utilized to integrate local air quality data with epidemiological and demographic information to quantify the health effects and associated economic values of poor air quality. A full description of the BenMAP program is provided in U.S. EPA (2023a,b).

The quantification of PM2.5-associated health effects is the product of several inputs including: (1) the change in air pollution due to coal trains; (2) the population exposed to varying levels of PM2.5 due to particulate dispersion; (3) the background prevalence or incidence of a given health endpoint such as asthma attacks or premature mortality and (4) the concentration-response functions (CRF) which relate the health risks associated with a given change in PM2.5, based on available epidemiological studies. The quantification of the health impact can be represented by:

ΔHE=Baseline*PopulationExposed*(1−exp(−β*ΔPM))

where ΔHE is the estimated increased in the health endpoint, Baseline is the background incidence or prevalence of the health effect, β (beta) is the health risk per μg/m^3^ based on the epidemiologic evidence and ΔPM is the designated change in PM2.5. The inputs used for the current study are discussed in detail below.

### 3.1. Exposures

This HIA evaluates the impact of an increase in PM2.5 associated with passing coal trains based on the study of Ostro et al. (2023a,b) in Richmond, CA (see map). To our knowledge, this is the only urban-based study of the impacts of coal trains. The study relied on artificial intelligence (AI) to train cameras to recognize different types of trains so that the specific impact of coal trains (versus freight or passenger trains) could be isolated. Since the schedule of coal trains is effectively random and not publicly available, the use of AI allowed for observation of thousands of trains passing through an observation point (Smithsonian Magazine, 2023).

The train monitor site was approximately 32 m from the rail line and relatively free of other sources of PM2.5: San Francisco Bay was to the west of the observation site, a park and golf course were situated to the east, and there were no major roads nearby. The increment in PM2.5 due to a passing train (either coal or freight) was determined by measuring concentrations during the train passage and comparing these to concentrations measured just prior to passage. Ultimately, increases in the 5-min average during the train passage were calculated for 15 full coal trains. Both coal and freight trains will produce both exhaust and non-exhaust PM2.5. By directly comparing the PM2.5 from passing coal trains versus that produced by passing freight trains, the basic result of Ostro et al. (2023a) found an increase of 8.32 μg/m^3^ which is strictly due to the coal dust.

However, the magnitude of the impact from the passing coal trains depends on meteorological conditions. Three bounding scenarios were identified, therefore, based on the effects of meteorology on pollutant dispersion. In additional analysis (Ostro et al., 2023b), the study reported an increment of 12.1 μg/m^3^ during relatively calmer wind days (roughly 50% of the time when windspeed was less than 3.1 m/s). During these days the nearby monitor was more likely to capture the full impact of the passing train. In another scenario, when the winds were calm and from the west (the dominant direction about 70% of the time) the statistical model indicated an increment of 25.0 μg/m^3^. Under these three scenarios, the 5-min increments were averaged with background concentrations of 10.4 in 2019 (the 3-year average at the closest EPA reference monitor in San Pablo) to obtain hourly increases in PM2.5 of 0.7, 1.0 and 2.1 μg/m^3^, respectively.

To translate this generic increment into specific population exposures, we estimated the annual number of coal trains that would pass the study population based upon a currently proposed coal export scenario in Oakland, CA. Developers have proposed shipping up to 10 million tons of sub-bituminous coal a year from Utah mines to a proposed export terminal in Oakland, CA (Tagami and Bridges, 2015). Current coal conveyance to an export terminal in nearby Richmond, California suggests that 2.6 million tons per year of the proposed coal (50,000 per week) would be loaded on a Panamax vessel in Stockton, CA, prior to reaching Oakland (National Coal Council, 2019). The remaining 7.4 million tons would arrive at the proposed Oakland port by rail.

The average coal train comprises 100 to 130 cars, each carrying about 100–130 tons (U.S. Energy Information Administration, 2023). Therefore, assuming 115 cars carrying 115 tons, the 7.4 million tons would require almost 10 trains per week. Since PM2.5 can remain in the air for hours to days, and also be re-entrained from passing freight trains, it is reasonable to assume a continuous chronic exposure to the train-generated PM2.5. Ultimately, a train-specific dispersion model estimate from Liu et al. (2022) was used to determine the likely dispersion pattern of the PM2.5 for the impacted cities in Contra Costa and Alameda counties. The spatial dispersion was developed from empirical observations and land use regression models based on 46 monitors located in the Los Angeles area. This study was unique in measuring the dispersion from passing trains.

### 3.2. Exposed population

Using the dispersion model described above, the resulting increment in PM2.5 was applied to the cities in the two counties in the Bay Area that would be impacted by the proposed coal trains traveling from Utah to Oakland, CA (see Fig. 1). Based on the 2010 U.S. Census, BenMAP provides population total and racial/ethnic composition at the census block level projected for the year 2023. Based on this information, the study area population that would experience some increase in annual PM2.5 totaled 262,031. Focusing on the two age groups considered most vulnerable to PM2.5, 14% of the total were over age 65 and 26% were under 18.

### 3.3. Baseline prevalence and incidence

BenMAP software package provides default levels of baseline prevalence and incidence. The assumptions and development for these levels are discussed in detail in EPA (2023 TSD). Baseline prevalence and incidence rates based on 2012–2014 data were projected from 2015 to future years, in five-year increments. This study employs prevalence and incidence rates for the year 2025, the closest year to our 2023 study available in BenMAP. We also examined prevalence and incidence rates specific to our study area to enable a comparison with those county-wide estimates provided by BenMAP.

### 3.4. Concentration-response functions

The CRF indicate the quantitative risk of each health endpoint per μg/m^3^, specified as a beta estimate, based on available epidemiological studies. In general, we relied on the defaults offered by the BenMAP software and by recent reviews conducted by U.S. EPA (2023a,b). However, for several endpoints, we utilized epidemiologic data that were more relevant, based on the concentration of PM2.5 in the study area, racial make-up, or improved exposure assessment and/or length of exposure to PM2.5. These changes are described below, and Table 1 summarizes the lead author and the population size and age range of those included for each health endpoint.

#### 3.4.1. Chronic exposure – all-cause mortality

Currently, there are many studies and meta-analyses of the all-cause mortality risk associated with chronic exposure to PM2.5. In addition, there are studies that have focused on the impacts at relatively low concentrations (PM2.5 < 12 μg/m^3^). The latter is more consistent with the concentrations observed in Richmond and Oakland, and much of the Bay Area. Among the more recent studies, Vodonos et al. (2018) conducted a meta-regression of 53 published studies and reported for their full hybrid model (a combination of satellite remote sensing, meteorology and land use as predictors, which resulted in a more accurate assessment of exposures), a risk estimate of 1.61% (95% CI = 1.18, 2.01) for a one μg/m^3^ change. For their model that was constrained to PM2.5 concentrations <10 μg/m^3^, each one μg/m^3^ increment contributed a mortality risk of 1.29% (95% CI = 1.06, 1.50). Pope et al. (2019) examined a cohort of 1.6 million individuals from the U.S. National Health Interview Surveys and reported an all-cause mortality risk of 1.22 (95% CI: 0.8, 1.5).

Chen and Hoek (2020) conducted a meta-analysis of 25 studies of all-cause mortality and reported an overall risk estimate of 0.8% (95% CI = 0.6, 0.9). This analysis was used as the basis for the World Health Organization Air Quality Guidelines. The authors also reported the risk for concentrations less than 12 μg/m^3^ of 1.2% (95% CI = 0.8, 1.7). Crouse et al. (2020) utilized mortality data for 2.4 million Canadian adults, ages 25–89 with relatively low concentrations of PM2.5. The study adds another dimension in that prior exposures of the previous 8 years were available, whereas most studies have exposure data for only a few years. Based on three-years of exposure data, the authors reported risk estimates of 2.0% (95% CI = 1.7, 2.3), for eight years of exposure data; the risk estimates were 2.3% (95% CI = 2.0, 2.7) for a one μg/m^3^ change. Finally, Chen et al. (2023) developed advanced exposure metrics and statistical approaches to examine large cohorts in the United States, Canada and Europe using a similar methodology. They reported a combined mortality risk of 0.8% (95% CI = 0.4, 1.2) for a one μg/m^3^ increment. In summary, the reported risks for these studies range from 0.8% to 2.1% for a one μg/m^3^ change, with the higher risks observed when concentrations <12 μg/m^3^ were examined.

Race- and income-specific mortality risk functions have also been developed. This information is relevant for the Bay Area cities impacted by rail traffic due to relatively high proportions of Black and Hispanic/Latino/a residents and pronounced income disparities in neighborhoods in this region. Specifically, 74% of the residents in the study area were non-White. Existing studies report much higher risk functions for people of color and for those of lower socioeconomic standing (Yazdi et al., 2021; Di et al., 2017; Pope et al., 2019; Ostro et al., 2008). For example, in a study of 61 million Medicare recipients, Di et al. (2017) reported risks of mortality associated with a one μg/m^3^ change in long-term average PM2.5 for White and Black persons of 0.6% and 2.1%, respectively. Yitshak-Sade et al. (2020) examined Black and White decedents from urban block-groups in Massachusetts. The authors reported that comparing blocks that were 1.5% Black to those that were 15% Black doubled the risk of PM2.5-associated mortality. In the Pope et al. (2019) study, the analysis by race reported the risks of mortality to Whites, Hispanics and Blacks of 1.1%, 2.0% and 1.55, respectively, for a one μg/m^3^. Finally, in a study of cardiovascular mortality among California residents, Ostro et al. (2008) reported the risk for Hispanic residents was more than double that of White residents.

Given some uncertainty as to the “best” mortality risk to apply in this study, a range of estimates are presented. As a first risk estimate, the results of Chen and Hoek (2020) with concurrence from Chen et al. (2023) of 0.8% is used and is labeled as “WHO Basic”. Since several of the cohorts used in this analysis had mean PM2.5 concentrations much above 12 μg/m^3^, as a second risk estimate, the Chen and Hoek (2020) results for studies less than 12 μg/m^3^ of 1.2% was used to match with historic concentrations in the Bay Area and labeled as “WHO <12 μg/m^3^” Finally, a third estimate, and one that is perhaps most relevant to the study population is adjusted for race and concentration less than 12 μg/m^3^. The studies above suggest an approximate doubling of the risk for people of color so the results of Di et al. (2017) for Black persons of 2.1% (95% CI = 2.0, 2.2) was used. This estimate is labeled “Race--Adjusted”. This information is likely relevant to a global context as well since rail lines and affiliated industrial activity typically transect neighborhoods that disproportionally house low income or ethnic minority people (Lane et al. (2022).

#### 3.4.2. Respiratory and cardiovascular hospitalization

Unlike the mortality studies which utilize information on annual or multi-year exposures, most of the morbidity endpoints in BenMAP are based on epidemiological studies that use daily or multi-day exposures. However, it is clear from the vast number of studies of the effects of PM2.5 on premature mortality that the risks of chronic exposure (one year or more) are typically 10 times or more than those associated with acute exposures of one or multiple days (see for example, U.S. EPA, 2019; 2023b; Kloog et al., 2012). Therefore, it is important to examine the impacts of chronic exposure on morbidity outcomes when data are available.

Fortunately, there are several studies that have examined the impact of chronic exposure to PM2.5 and hospital admissions for various disease endpoints. For example, Yazdi et al. (2019) determined the marginal effect of chronic exposure to PM2.5 on first hospital admissions using Medicare data of 11 million participants from seven southeastern states in the United States. A highly refined model was developed to estimate PM2.5 using data from satellite remote sensing, land use and chemical transport models over a 13-year period. The relevance and use of the study for our health impact assessment was enhanced by their sensitivity analysis which examined the risks for concentrations less than 12 μg/m,^3^ generally in the same range as in our study area. They reported the following risks of hospitalization for a change of one μg/m^3^ of PM2.5: stroke (5.2%), COPD (7.3%), heart attack (3.4%), pneumonia (10.1%) and heart failure (7.6%).

The findings for cardiovascular disease are supported by at least two other studies on long-term exposure and hospitalization (Miller et al., 2007; Wang et al., 2023). For example, Miller et al. (2007) utilized data from the Women’s Health Initiative Observational Study, a prospective cohort study with PM2.5 measured over a six-year period, on average. The outcome examined was first cardiovascular event, which included first occurrence of either myocardial infarction (MI), coronary revascularization, stroke, and death from either coronary heart disease or cerebrovascular disease. Overall, a one μg/m^3^ change in PM2.5 was associated with a 2.4% (95% CI = 0.9, 4.1) increase in a cardiovascular event. When within-city differences in PM2.5 were analyzed, the risks were estimated at 6.4% (95% CI = 2.4, 11.8). Wang et al. (2023) estimated the relationship between annual exposure to PM2.5 and hospitalizations for 10 US states over a 15-year period. In total, almost 2 million MI hospital admissions from 8,106 zip codes were included in this study. The results indicated that when considering only areas where exposures were always less than 12 μg/m^3^ over the study period, the risks were 2.17% (95% CI = 1.79, 2.56) for a one μg/m^3^ change.

These relatively consistent studies provide support for the use of long-term exposures in our assessment. For hospital admissions, therefore, we utilize the findings of Yazdi et al. (2019) since they encompass a suite of health outcomes associated with PM2.5.

#### 3.4.3. Asthma medication

Given the high rates of asthma in the cities of Oakland and Richmond (BAAQMD, 2018), it is important to examine the effects of PM2.5 on asthma exacerbation and the need for rescue medication. Rabinovitch et al. (2006) studied a school-based cohort of 73 children followed daily over two winters in Denver, Colorado. Data were collected on peak concentrations of PM2.5 in the morning along with concurrent use of a bronchodilator medication, albuterol. The study reported a 2.6% increase in medication associated with a 12 μg/m^3^ change in peak PM2.5, which was converted to a beta of 0.002 per μg/m^3^. We then utilized the same dispersion patterns as described earlier to determine the increase in medication as a proxy for days with asthma exacerbation.

## 4. Results

The health impacts were quantified for the three different scenarios described earlier that are based on differing conditions regarding wind speed and direction. Ultimately, the scenarios generated increases in annual average PM2.5 of 0.7, 1.0 and 2.1 μg/m^3^. Table 2 summarizes the estimated health impacts for the 262,000 people who are exposed to an increase in PM2.5 from the passing coal trains. Although this is a relatively small population, it is a microcosm of what likely occurs to millions of urban dwellers throughout the world as uncovered coal trains traverse the globe delivering coal to power plants and export terminals.

Focusing on the most severe outcomes for this study population, across the three exposure scenarios the annual mortality impacts of coal trains range from 2 to 6 deaths per year in the basic model and from 5 to 15 in the race-adjusted model. Hospital admissions for cardiovascular disease and pneumonia range from 9 to 28, and 6 to 17, respectively, over the three scenarios. For asthma, over the three scenarios, new cases range from 7 to 22, while asthma symptoms range from 19,000 to 58,000 days attributable to coal train transit. With asthmatics making up 6.4% of the 6 to 17 age group, this means an average of about 10 symptom days per year per asthmatic. These estimates amount to increases of between one and six percent over baseline levels.

Based on the middle scenario of a one μg/m^3^ change in annual average, there is an estimated increase per year of three (basic model) to seven deaths (race-adjusted model), five hospital admissions for chronic lung disease, nine for congestive heart failure, eight for pneumonia, six for stroke and 14 for all cardiovascular disease. The analysis also indicates about 11 new cases of asthma and 27,000 additional asthma symptoms requiring medication. Among the less severe outcomes, 1,300 additional cases of hay fever/rhinitis, 3,300 days with some restrictions in activity and 560 additional days of work loss are estimated.

Table 3 summarizes the analysis of relative PM2.5 increments by race/ethnicity. The results indicate that Hispanic and Black residents in the study area have a 41% and 29% higher level of exposure to PM2.5, respectively, relative to White residents.

## 5. Discussion

Our study is the first to estimate the health impacts of uncovered passing coal trains. The central finding is that exposures to PM2.5 from these trains are associated with significant mortality and morbidity. While the absolute number of cases is relatively small due to the size of the catchment area, under certain scenarios they amount to increases of one to six percent over baseline. Further, these findings provide a microcosm of what might be expected globally given the multiple millions that are exposed by the common traverse of coal trains. This study based its parameters on a specific – though generalizable – coal export proposal and concludes that the anticipated number of weekly mile-long trains that would be delivering coal to the proposed terminal will result in chronic exposure to elevated PM2.5. The quantification of subsequent health impacts is noteworthy for finding previously unrecognized and severe impacts such as death and hospitalization and for including the addition of several morbidity outcomes associated with long-term exposures.

For example, in the worst-case scenario of a 2.1 μg/m^3^ increase near the rail line, premature mortality would increase by 1.3% while hospital admissions for chronic lung disease, pneumonia and cardiovascular disease would increase by 4.7%, 6.2% and 2.2%, respectively. In addition, the number of new cases of asthma and the number of days of asthma exacerbation would increase by about 3.2%. These percent increases are reduced by half and two-thirds in the 1.0 μg/m^3^ and 0.7 μg/m^3^ scenarios, respectively.

While the long-term exposure impacts are noteworthy, it is important to note that there is epidemiological evidence of additional impacts after exposures as short as one or more hours. This is relevant due to the high peaks (5-min average) of PM2.5 observed at the rail line, The sub-acute impacts of PM2.5 on asthma exacerbation was included in above estimates. However, there are also other studies indicating that exposures as short as 1 h (or a few hours) can increase the risk of outcomes such as acute MI, arrhythmia, hospitalization and emergency department visits for cardiovascular and respiratory disease and ambulance calls (Peters et al., 2004; Kim et al., 2015; Chen et al., 2022; Wu et al., 2020).

For several reasons, the impacts in this assessment are likely underestimated. First, several important health impacts either are not included in BenMAP or were not estimated due to lack of sample size in the study population. These include adverse birth outcomes (i.e., pre-maturity, low birthweight), neurological impacts on infants and cognitive impacts on adults (i.e., depression, Parkinson’s and Alzheimer’s diseases) and lung and other organ cancers. Second, we have not estimated health impacts associated with exposures to ultrafine or coarse particles from the passing trains.

Third, the baseline rates of mortality, heart disease and asthma are higher in the study area than those used in the BenMAP software, which are county-level data. For example, data from the Alameda County Public Health Department shows that in West Oakland (the site where the proposed terminal would be located and where trains would pass through), the rates of asthma emergency room visits and hospitalizations, heart disease and stroke mortality, and cancer rates are all about twice the county average. The Department also confirms that the Oakland population living within one mile of rail lines is markedly different demographically than that living farther away, with a higher percentage of people of color, children, and adolescents, and people living in poverty (ACPHD, 2016).

Likewise, for the study population located in Richmond, several of the census tracts abutting the rail line have mortality rates 10%–50% higher than the county average. The higher risk and baseline levels for the study population relative to county estimates would obviously result in higher quantitative estimates of the health impacts. Finally, the estimates do not include the potential PM2.5 exposures and impacts of loading and unloading at the proposed terminal itself.

There are also several limitations and uncertainties in the assessment. First, the original estimates of the impact of passing uncovered coal cars are based on only three studies, although the results of the two larger studies are very consistent (Ostro et al. 2023a, 2023b; Jaffe et al., 2015). Second, due to lack of available studies that specifically measured the dispersion of PM2.5 from trains, we relied on a single study for the dispersion model. However, the actual measurement of PM2.5 concentrations in that study often provides a better measure than dispersion models based on emissions factors. Interestingly, in this case, our findings for the increments are similar to those provided by the dispersion model for coal trains developed by Grey (2017). Third is the assumption of equal toxicity amongst the components of PM2.5, a common assumption in most impact assessments including the Global Burden of Disease (Fuller et al., 2022).

Taken together, our estimates provide a reasonable assessment of the likely adverse health effects of uncovered coal cars traversing through urban areas throughout the world. They indicate that coal’s impacts, including mortality, extend beyond coal combustion and mining to its rail conveyance and export. Given that the three largest users of coal are India, China, and the U.S., and that over 80 countries use coal, this study confirms that large populations are impacted by coal trains, making this practice a heretofore insufficiently studied or regulated public health concern of global proportions.

Unfortunately, there are limited possibilities for mitigation of the particle emissions of passing coal trains. Traditionally, chemical surfactants have been applied to the coal piles, but they tend to degrade over time and space. Coal car covers are not commercially available and if used could increase the risk of spontaneous combustion since coal is exothermic. In addition, even if available, the covers would render the coal cars heavier, leading to the risk of impairment of the train tracks and potential derailment.

That the young and old, low income, and communities of color are disproportionately burdened by heightened exposure and vulnerability to rail transport of coal as well as to the climate impacts of its combustion indicate that the matter of coal train activity also constitutes a matter of environmental justice.

## Figures and Tables

**Table 1:** Health endpoints, studies and populations used in the analysis.

Endpoint	Author	Start Age	End Age	Population
Mortality, All Cause	Chen and Hoek (2020)	25	89	178639
Mortality, All Cause, PM2.5 < 12	Chen and Hoek (2020)	25	89	178639
Mortality, All Cause, Race Adjusted	Di et al. (2017)	25	89	178639
HA Chronic Lung Disease^a^	Yazdi et al. (2021)	65	99	36139
HA Congestive Heart Failure	Yazdi et al. (2021)	65	99	36139
HA Pneumonia	Yazdi et al. (2021)	65	99	36139
HA Stroke	Yazdi et al. (2021)	65	99	36139
HA All Cardiovascular	Yazdi et al. (2021)	65	99	36139
Acute MI, Nonfatal	Peters et al. (2004)	65	99	36139
Asthma Symptom Days #	Rabinovitch et al. (2006)	6	17	35265
New Asthma	Tetreault et al. (2016)	0	4	18039
New Asthma	Tetreault et al. (2016)	5	17	38375
Hay Fever/Rhinitis	Parker et al. (2009)	3	17	45555
Minor Restricted Activity Days	Ostro and Rothschild (1989)	18	64	169487
Work Loss Days	Ostro (1987)	18	64	169487

Note: HA = Hospital admissions; MI = Myocardial Infarction; ER = Emergency Room visits.

a Not including asthma,

# Based on albuterol use.

**Table 2:** Estimated Health Effects and Associated Confidence Intervals due to passage of coal trains.

Endpoint	Estimated Cases for Alternative Peak Annual Average Increments (μg/m3)		
	0.7	1.0	2.1
Mortality, WHO Basic	2.0 (1.6, 2.4)	2.8 (2.4, 3.2)	5.9 (4.8, 6.8)
Mortality, WHO <12 μg/m^3^	2.8 (2.3, 3.2)	3.9 (3.2, 4.6)	8.2 (6.7, 9.6)
Mortality, Race-Adjusted	5.1 (4.2, 6.0)	7.3 (6.0, 8.5)	15.2 (12.5, 17.8)
HA Chronic Lung Disease^a^	3.5 (3.4, 3.7)	5 (4.8–5.2)	10.4 (10–10.8)
HA Congestive	6.4 (6.1–6.6)	9.1 (8.7–9.4)	18.8 (18–19.5)
HA Pneumonia	5.9 (5.6–6.1)	8.3 (8–8.6)	17.1 (16.5–17.8)
HA Stroke	4.1 (3.6–4.7)	5.9 (5.1–6.6)	12.2 (10.5–13.8)
HA All Cardiovascular	9.4 (8.3–10.5)	13.4 (11.8–15)	28 (24.6–31.1)
Acute MI, Nonfatal	1.1 (0.2–1.8)	1.5 (0.3–2.6)	3.2 (0.7, 5.5)
Asthma Symptom Days^b^	19.3 (6.7, 31.0)	27.0 (19.5, 43.3)	57.6 (201, 91.9)
New Asthma (age 0–4)	4.4 (4.3–4.6)	6.3 (6.1–6.6)	13.2 (12.6–13.7)
New Asthma (age 5–17)	3.1 (2.9–3.2)	4.4 (4.2–4.5)	9 (8.7–9.4)
Hay Fever/Rhinitis	45 (11–78)	64 (15–111)	134 (33–231)
MRAD	2.3 (1.9–2.7)	3.2 (2.7–3.9)	6.9 (5.6–8.1)
Work Loss Days	395 (333–455)	564 (476–650)	1184 (998–1363)

Note: HA = Hospital admissions; HF = Heart Failure; RAD = Restricted Activity Days.

a Not including asthma

b In thousands

**Table 3:** Study Population and Associated Increment in annual average PM2.5

	Hispanic	Asian	Black	NatAmer	White	Total
Population	90686	44649	57334	845	68525	262039
% Population	34.6%	17.0%	21.9%	0.3%	26.2%	100.0%
Annual Average PM2.5 Increment	0.82	0.62	0.75	0.67	0.58	0.71
Relative PM2.5 Levels	1.15	0.88	1.07	0.95	0.81	1.00

## Data Availability

Data will be made available on request.
